# The influence of TLR4 signaling on retinal ganglion cell survival and angiogenic response in a mouse model of oxygen-induced retinopathy

**DOI:** 10.1016/j.bbrep.2026.102550

**Published:** 2026-03-17

**Authors:** Yasunari Munemasa

**Affiliations:** Department of Ophthalmology, St Marianna University School of Medicine, 2-16-8 Sugao Miyamaeku, Kawasaki City, Kanagawa, 216-8511, Japan

**Keywords:** Retina, Retinopathy of prematurity, Toll-Like Receptor 4 (TLR4)

## Abstract

**Purpose:**

Retinopathy of prematurity (ROP) is a critical concern in neonatal care and potentially leads to vision impairment. Despite advancements in anti-VEGF treatments, the mechanisms driving pathological vitreoretinal neovascularization remain unclear. I examined the role of Toll-like receptor 4 (TLR4) in modulating inflammatory cytokines, angiogenesis, and neuronal cell protection in a mouse model of oxygen-induced retinopathy (OIR).

**Materials and methods:**

C57BL/6J TLR4−/− mice were subjected to OIR by exposure to 75% oxygen from postnatal days 7 to 12 (P7 to P12) following approved protocols. I used immunohistochemistry to assess TLR4 expression at P19, real-time quantitative PCR for proinflammatory cytokines at P19, ex vivo fluorescent vascular imaging to evaluate retinal vascular changes at P19, and retinal neuronal cells death evaluated by whole-mounted retina stained with cresyl violet at P47. Statistical significance was determined using one-way ANOVA (p < 0.05).

**Results:**

Immunofluorescence demonstrated TLR4 expression in microglia in OIR retinas of wild-type mice but not in controls. Real-time PCR revealed significant upregulation of vascular endothelial growth factor (VEGF) and monocyte chemoattractant protein-1 (MCP1) in OIR retinas, which was mitigated in TLR4−/− mice. Retinal angiogenesis significantly increased in wild-type OIR mice, whereas TLR4 knockdown inhibited these changes. Additionally, OIR caused approximately 30% neuronal cell death in the retinal ganglion cell layer, which was largely prevented in the TLR4−/− mice.

**Conclusions:**

These findings underscore TLR4's pivotal role in the regulation of inflammatory responses and angiogenesis in ROP. Targeting TLR4 may represent a novel therapeutic approach to preserve retinal integrity and improve visual outcomes in at-risk populations, particularly in premature infants.

## High light

• TLR4 modulates inflammation & angiogenesis in oxygen-induced retinopathy (OIR).
• TLR4 knockout suppresses VEGF/MCP1 upregulation in OIR mouse retina.
• Knockdown of TLR4 inhibits retinal neovascularization in the OIR model.
• TLR4 deficiency protects retinal ganglion cells from death in OIR.
• Targeting TLR4: a novel approach to treat retinal disease in premature infants.

## 1 Introduction

With recent developments in the management of premature infants, the incidence of retinopathy of prematurity (ROP) remains a significant concern in clinical practice. Although advancements in anti-VEGF treatments have improved visual prognosis, many cases continue to present treatment challenges [1]. Considerable scientific and clinical efforts have been focused on identifying the mechanisms of vascular injury that lead to pathological vitreoretinal neovascularization (NV). Recent studies have suggested that local neurons and glial cells, particularly microglia, also play critical roles in the pathological growth of blood vessels [2,3].

Neuronal cell death is an irreversible process that severely impacts visual outcomes, making it vital to control neurodegenerative changes in ROP. The literature robustly documents that intravitreal injection of small amounts of tumor necrosis factor (TNF) can induce axonal degeneration and subsequent retinal ganglion cell (RGC) death, with active microglia implicated in these mechanisms [4,5]. Therefore, microglial activity is linked not only to angiogenesis but also to inflammation-induced neuronal death, particularly through TNF-mediated pathways.

Toll-like receptor 4 (TLR4) serves as a key receptor for a variety of ligands, such as lipopolysaccharides, initiating the activation of microglia and the release of harmful factors that may compromise both vascular and neuronal integrity [[6], [7], [8], [9]].

Recent studies have underscored TLR4's involvement in promoting neovascularization within ischemic neural tissues and its crucial role in mediating ischemia-induced retinal damage through microglial activation [10,11]. Consequently, targeting TLR4 holds promise for providing protective benefits to both vascular and neuronal cells.

Due to the long-term complications associated with ROP, such as glaucoma and retinal detachment [12], there is a pressing need to focus on neuroprotective strategies that can safeguard vision in affected premature infants. My primary hypothesis posits that the modulation of TLR4 significantly influences inflammatory processes and angiogenesis during oxygen-induced retinopathy. In the present study, I investigated the effects of TLR4-mediated changes in inflammatory cytokines on both angiogenesis and neuronal cell protection within the mouse retina.

## 2 Methods

### 2.1 OIR model

All animal experiments were conducted in accordance with the ARVO Statement for the Use of Animals in Ophthalmic and Vision Research, and were approved by the Animal Research Committee of St. Marianna University School of Medicine (TG140730-3). This study adhered to the principles outlined in the National Research Council's Guide for the Care and Use of Laboratory Animals. Male C57BL/6J TLR4−/− mice were purchased from Oriental BioService Adults (Kyoto, Japan). The mice used in this study had an average weight of 10-15 g at P19 and 20-30 g at P47. The animals were housed in a controlled environment with fluorescent lighting (330 lx) and a 12-h light/dark cycle at a temperature of 21 °C.

Oxygen-induced retinopathy (OIR) was performed as previously reported [13]. Briefly, newborn mice were exposed to 75% oxygen from postnatal days P7 to P12. To minimize litter-to-litter variability, pups from each litter were randomly assigned to either the normoxia (normal room air) or OIR group for comparative analysis. A minimum of three litters was used per experimental group. To ensure reliable data, all the experimental groups included age- and sex-matched controls. For retinal PCR experiments (n = 5), each sample contained two retinas collected from two different mice, derived from a minimum of three litters. The primary endpoints for analysis were set at P19 and P47 to evaluate the distinct phases of OIR pathology. Mice were euthanized at P19 to investigate the vascular remodeling phase, which follows the peak of pathological neovascularization. This time point was selected to assess the persistence of pathological vessels and the early stages of physiological revascularization through gene expression studies, immunohistochemistry (IHC), and FITC-labeled angiography. Furthermore, mice were euthanized at P47 to evaluate long-term neurodegeneration. Since RGC loss and inner retinal thinning in the OIR model are stable and quantifiable after the regression of pathological vascular tufts, the P47 time point ensures the measurement of permanent neuronal loss rather than transient cellular stress. Histological studies using cresyl violet staining were performed at this stage to quantify RGC survival.

### 2.2 Immunohistochemistry

To assess the localization of TLR4 protein, double immunofluorescence was performed on retinal sections. Mice were anesthetized with a mixture of ketamine and xylazine (10 and 5 mg/kg, respectively), and then perfused transcardially with 4% paraformaldehyde in phosphate buffer (0.1 M). The eyes were enucleated at P19, and 5-μm-thick sections were prepared.

Sections were incubated with primary antibodies against TLR4 (1:100 dilution, SantaCruz Biotechnology, Santa Cruz, CA) and Iba-1 (1:50 dilution, WAKO, Osaka, Japan) overnight at 4 °C. The incubation conditions (time and temperature) were optimized to enhance specificity. Following primary antibody incubation, the sections were processed with appropriate secondary antibodies for FITC-conjugated anti-rabbit IgG or rhodamine-conjugated anti-donkey IgG (Cappel Research Products, Durham, NC, USA) for visualization.

Evaluation of Non-Specific Binding: Sections were processed without primary antibodies to evaluate non-specific binding. The absence of a signal in these sections demonstrated the lack of tissue autofluorescence and non-specific staining from the secondary antibody, indicating that any observed fluorescence in the experimental samples was likely due to specific binding of the primary antibody to TLR4.

### 2.3 Real-time Polymerase Chain Reaction (PCR)

Real-time quantitative reverse-transcription PCR was performed using an Applied Biosystems protocol (Life Technologies, Tokyo, Japan). Retinas were collected at P19 from two mice per sample, and then total RNA was isolated using ReverTra Ace (TOYOBO, Osaka, Japan). The expression of β-actin was used as a reference gene for normalization to account for variability in total cDNA levels across samples. While the use of multiple reference genes is becoming increasingly common, β-actin remains a widely used normalization standard in retinal research due to its consistent expression profile under many experimental conditions.

Specific primer sequences for the target genes (VEGF, ICAM-1, TNF-α, IL-1β, TGF-β, and MCP-1) and the reference gene (β-actin), along with their amplicon sizes and GenBank Accession Numbers, are detailed in Table 1.

**Table 1:** Primer sequences used for quantitative PCR.

Gene Name	Forward Primer (5' → 3′)	Reverse Primer (5' → 3′)	Amplicon Size (bp)	GenBank Accession No.
VEGF	ACTGGACCCTGGCTTTACTG	TCTGCTTCCCCTTCTGTCGT	78	BC168708.1
ICAM-1	AAACCAGACCCTGGAACTGCAC	GCCTGGCATTTCAGAGTCTGCT	111	NM_010493
TNF-α	CTACTCCCAGGTTCTCTTCAA	GCAGAGAGGAGGTTGACTTTC	111	NM_001278601.1
IL-1β	CGAGGCTAATAGGCTCATCT	GTTTGGAAGCAGCCCTTCAT	710	XM_006498795
TGF-β	CTCCCGTGGCTTCTAGTGC	GCCTTAGTTTGGACAGGATCTG	133	NM_011577.2
MCP-1	GATCTGTGCTGACCCCAATAAGG	GGTGCTGAAGTCCTTAGGGTTGA	123	NM_001286598.1
β-actin	AACACCCCAGCCATGTAC	ATGTCACGCACGATTTCCC	254	NM_007393.5

To validate the specificity of the amplification, standard curves were generated from serial dilutions of known template concentrations. Negative controls without template were included in each run to verify the absence of contamination. Results are expressed as fold-changes relative to wild-type mouse retina controls under normoxic conditions.

### 2.4 Retinal angiogenesis investigated by FITC-dextran heart perfusion and retinal preparation

Retinal vascular angiogenesis was evaluated using fluorescein angiography. Mice at P19 were anesthetized and a surgical approach was used to clamp the abdominal aorta above the liver. Following the opening of the right auricle, 40 μg/ml FITC-dextran (Sigma-Aldrich, St. Louis, MO, USA) were injected into the left ventricle. After a 5-min circulation period, the retinas were flat-mounted and observed under a fluorescence microscope.

Angiogenesis was evaluated using whole-mount photographs of the retina, which were converted to 8-bit grayscale images using FIJI (https://fiji.sc). To quantify angiogenesis, I selected a standardized region of interest (ROI) from the entire retina. The mean gray value (intensity) within this ROI was measured using FIJI. This mean gray value is inversely proportional to the amount of FITC-dextran present, with lower values indicating greater vascular density and thus, more angiogenesis.

I acknowledge that while this intensity-based approach provides valuable data, it initially included normal blood vessels, which may not accurately reflect changes in pathological neovascularization. Therefore, I ensured that the vascular assessments clearly distinguish physiological vascular regrowth/revascularization from pathological neovascularization (NV).

To clarify the depiction of neovascularization, I performed automatic measurements of vascular density and graphically represented the proportion of neovascular tufts per retinal area. While FIJI automates the measurement of vascular area, the selection of vascular tufts is a manual process, which could impact reproducibility. To address this, I conducted each measurement three times and calculated the average for improved accuracy.

This intensity-based approach allows for an objective and quantitative assessment of neovascularization, and I adjusted the methodology in accordance with prior validated methods [14].

### 2.5 Morphometry of cells in the Ganglion Cell Layer (GCL)

Morphometric analysis of neural cells in the GCL from whole-mounted (flat-mounted) retinas was conducted as previously described [15]. Eyes were obtained at P47, a timepoint chosen because I hypothesized that molecular changes related to inflammation and cell death would occur earlier, followed by gradual neurodegeneration detectable at P47 and retinas were stained with 1% cresyl violet, a metachromatic basic dye that stains nucleic acids, allowing for visualization of Nissl substance in neuronal cell bodies. Counts of all retinal ganglion cells (RGCs) and amacrine cells in the retinal ganglion cell layer (RGCL) were performed manually by a single masked observer in eight predetermined areas, specifically at distances of 500 and 1000 μm from the optic disc in each retinal quadrant (nasal, temporal, inferior, and superior). Neuronal cell bodies were anatomically identified as being in the RGCL by adjusting focus through the depth of the tissue in these areas. To ensure accurate identification, I specifically looked for cells with a clearly defined nucleus and prominent nucleolus within a distinct cell body and excluded any ambiguously stained or fragmented cells. Counting was performed using a standard light microscope with a 10x eyepiece and a 100× oil immersion objective.^14^ To minimize observer bias, quantification was performed in a blinded manner.

### 2.6 Statistical analysis

Data are presented as the mean ± SEM. Differences among groups were analyzed using one-way ANOVA, followed by Scheffe's method or Mann–Whitney's method as appropriate. A probability value of less than 0.05 was considered statistically significant. Analyses were conducted using Stat Plus (AnalystSoft Inc., Alexandria, VA) version 8.0.3 to ensure the robustness methods and adequately addresses the complexities of data.

## 3 Results

### 3.1 TLR4 expression in P19 OIR retinas of wild-type mice

**Fig. 1:**
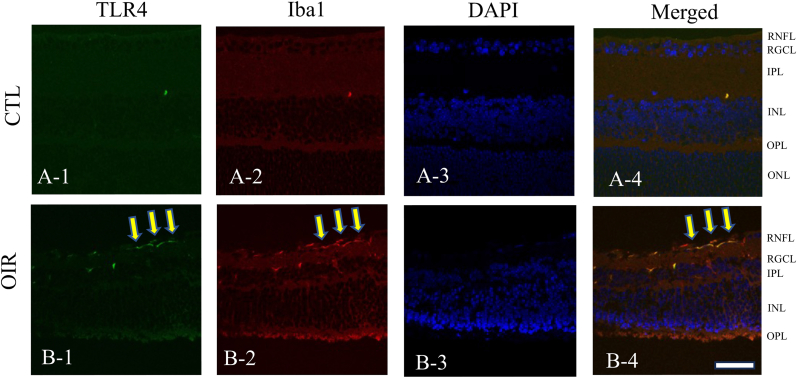
**Immunohistochemistry** TLR4 localization was confirmed by immunohistochemistry with an Iba1 antibody. A-1 to A-4 represent the retina of the control P19, while B-1 to B-4 represent the retina of the OIR P19 (Fig. 1 A-1-A-4). TLR4 was localized to Iba1, a microglia, in theP19 OIR retina (Fig. 1 B-1-B-4). RNFL; Retinal Nerve Fiber Layer, RGCL; Retinal Ganglion Cell layer, IPL; Inner Plexiform Layer, INL; Inner Nuclear Layer, OPL; Outer Plexiform Layer, ONL; Outer Nuclear Layer. Scale bar = 100 μm.

Double immunofluorescence showed that colocalization of TLR4 was observed in Iba-1 positive cells, a marker for microglia, in the inner retina at P19 OIR of wild-type mice (Fig. 1B-∼B-4). In contrast, no TLR4 expression was found in the control retinas of wild-type mice (Fig. 1A-∼A-4).

Due to the near absence of TLR4 staining in the control retinas, I determined that a quantitative comparison of TLR4 expression would not provide meaningful additional information beyond the clear qualitative difference observed between the OIR and control groups. The induction of TLR4 in microglia following OIR is readily apparent by immunohistochemistry.

### 3.2 Changes in pro-inflammatory cytokine levels in the P19 OIR retina

**Fig. 2:**
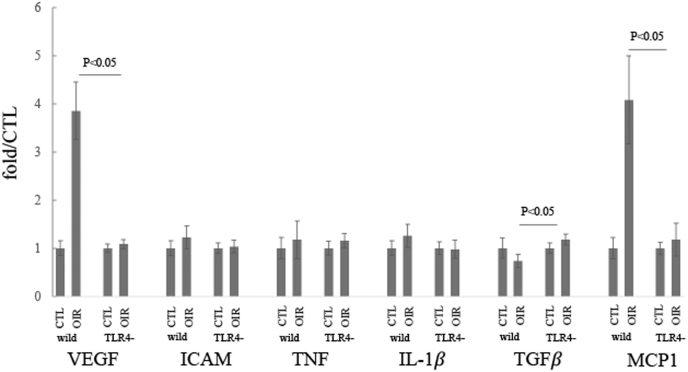
**Real time PCR** Real-time PCR was performed using primers for VEGF ICAM-1, TNF, IL-1β, TGFβ, and MCP1. The changes observed in the real-time PCR analysis of wild-type mice during oxygen-induced retinopathy (OIR) were compared with those in the wild-type control (naive) retina at P19 (n = 5). Similarly, changes in TLR4−/− mice during OIR were compared to those in the TLR4−/− control (naive) retina (n = 5).

Real-time PCR was used to study the changes in *the mRNA levels* of pro-inflammatory cytokines, such as VEGF, ICAM-1, TNF, IL-1β, TGF-β, and MCP1, in CTL and OIR retinas. Dramatic upregulation of VEGF and MCP1 mRNA was observed in the OIR of wild-type mice retina, compared to CTL, approximately increased fourfold, respectively (Fig. 2C). No significant changes in mRNA levels of ICAM-1, TNF, IL-1β, and TGF-β were observed between the CTL and OIR retinas of wild-type mice. This dramatic upregulation of VEGF and MCP1 *mRNA* was not observed in the OIR retinas of TLR4−/− mice (Fig. 2) (n = 5, p < 0.05).

### 3.3 Retinal angiogenesis in OIR retinas

**Fig. 3:**
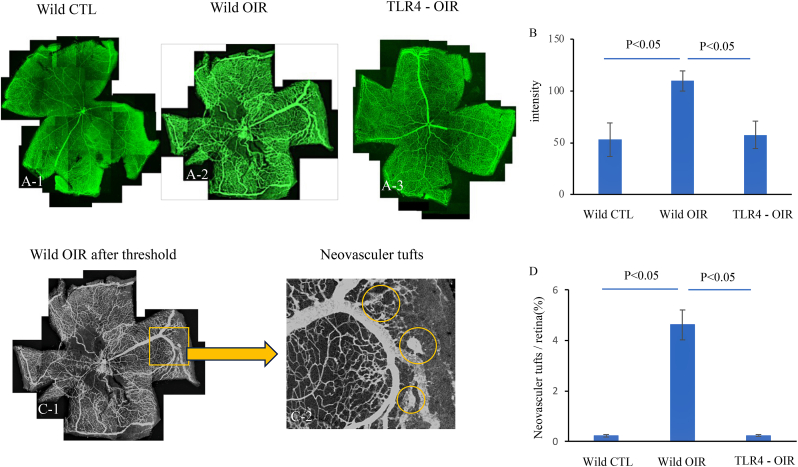
**Retinal angiogenesis** Whole-mount retina of FITC-labeled angiography for mice of P19 wild control (Figs. 3 A–1), P19 OIR of wild-type mice (Figs. 3 A–2), and TLR4 −/− mice (Fig. 3 A-3). Quantification of the intensity of the blood vessels and ratio of neovascular tufts was performed using ImageJ software (n = 4). The graph illustrates the measured intensity levels across the different regions of interest within the retinal images (Fig. 3 B). Neovascular tufts were expressed as a ratio to the retina and graphically represented (Fig. 3 C).

Retinal angiogenesis was distinctly observed in the OIR retina of wild-type mice with P19 compared to the control retina (without hypoxia) (Figs. 3A–1 and A-2). The knockdown of TLR4 completely inhibited OIR-induced changes in angiogenesis (Fig. 3A-). Analysis and quantification of vessel intensity and neovascular tufts using FIJI (Figs. 3C–1 and 3C-2) showed that the OIR significantly increased in wild-type mice compared to that in control mice (n = 4, p < 0.05), whereas this elevation was suppressed in TLR4−/− mice (n = 4, p < 0.05) (Fig. 3B and D). These changes were more prominently observed in neovascular tufts than in intensity (Fig. 3D).

### 3.4 Retinal neuronal change in OIR

**Fig. 4:**
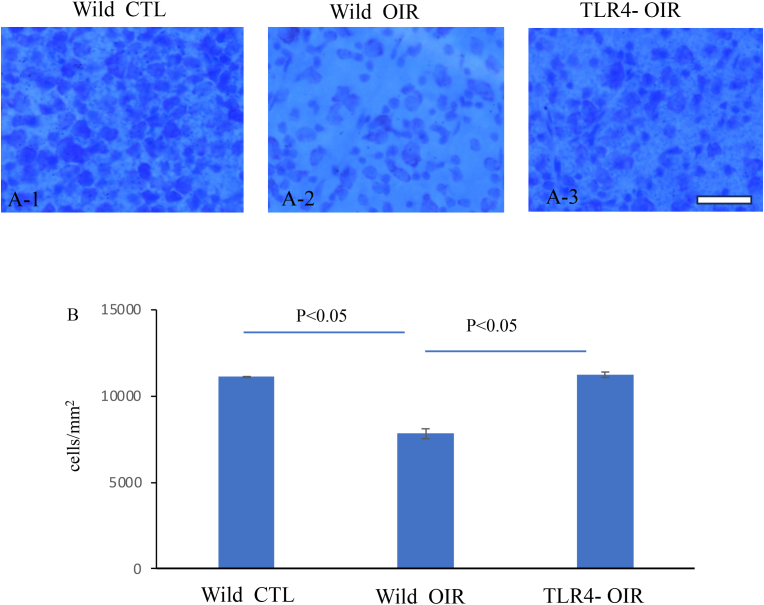
**Cresyl violet staining** The neuronal cells in the RGCL were stained with cresyl violet for P47 wild-type control mice (Figs. 4 A–1), P47 OIR in wild-type mice (Figs. 4 A–2), and P47 OIR in TLR4 −/− mice (Figs. 4 A–3). A quantitative graph showing the number of neuronal cells in the RGCL of whole-mount retinas stained with cresyl violet is presented (Fig. 4 B) (n = 6). Scale bar = 100 μm.

Cresyl violet staining for flat preparation of the retina was performed to study neuronal cell damage induced by OIR. OIR treatment led to approximately 30% neuronal cell death within the retinal ganglion cell layer (RGCL) (n = 6, p < 0.01) (Fig. 4A and B). TLR4 knockdown almost completely suppressed neuronal cell death (n = 6, p < 0.05) (Fig. 4A and B).

## 4 Discussion

The findings presented in this study provide valuable insights into the role of Toll-like receptor 4 (TLR4) in the development of retinopathy of prematurity (ROP). Our results indicate that TLR4 signaling influences angiogenesis and promotes ganglion cell survival protection in this model. Further investigation will be required to elucidate the precise mechanisms by which TLR4 mediates these effects, including a more comprehensive analysis of inflammatory cytokine profiles and downstream signaling pathways.

Consistent with prior research, I observed upregulation of pro-inflammatory cytokines, particularly vascular endothelial growth factor (VEGF) and monocyte chemoattractant protein-1 (MCP1), in the retinas of wild-type mice. Previous studies have shown that TLR4 activation can initiate an inflammatory cascade leading to aberrant vascular growth and neurodegeneration [16,17]. It is important to note that while some inflammatory markers, such as ICAM-1, TNF, IL-1β, and TGF-β, did not show statistically significant changes in our analysis at P19, the significant suppression of the VEGF/MCP-1 axis in TLR4−/− mice provides strong evidence for a mechanistic link. In the OIR model, VEGF is the primary driver of pathological neovascularization, and MCP-1 is essential for the recruitment of activated microglia and macrophages. The lack of significant alterations in other cytokines may be attributed to the timing of our analysis; inflammatory markers like TNF and IL-1β often exhibit rapid and transient expression peaking shortly after the transition to normoxia (around P12–P14). By P19, the peak phase of neovascularization, these acute responses may have subsided, whereas the VEGF-driven angiogenic response remains prominent. Furthermore, our immunohistochemical data showing the specific colocalization of TLR4 with activated microglia—nearly absent in normoxia—suggests that TLR4-mediated inflammation is highly localized and cell-specific. This targeted microglial activation is likely sufficient to drive pathological processes through key mediators like VEGF and MCP-1, even in the absence of a generalized increase in all systemic inflammatory markers.

Regarding the specific downstream signaling pathways linking TLR4 to VEGF/MCP1 regulation, I previously demonstrated in a different retinal injury model that TLR4 signaling directly regulates Mitogen-Activated Protein Kinase (MAPK) pathways. In our previous study using intravitreal injection of Histone H2B, a known TLR4 ligand, I proved that TLR4 deficiency significantly suppresses the dephosphorylation of *p*-ERK and inhibits the phosphorylation of p38 and JNK, thereby exerting a neuroprotective effect [15]. The results from this previous publication, underscores the role of TLR4 as a critical mediator of these intracellular signaling pathways. Although the OIR model is distinct, both models share a common pathological basis in inflammation-mediated neuronal death. Therefore, it is highly probable that the MAPK signaling cascade is a key mechanism through which TLR4 mediates the expression of VEGF and MCP1 and subsequent neuronal loss in ROP [18,19].

Importantly, Chen et al. demonstrated in HRMECs that TLR4 activation upregulates MAP4K4 signaling, promoting cell proliferation and migration. This highlights a potential VEGF-independent pathway regulated by TLR4 that contributes to retinal neovascularization [20]. Chen et al. also showed that inhibition of TLR4 with TAK-242 reduced areas of nonperfusion and inhibited aberrant angiogenesis in the OIR model. These results suggest a promising therapeutic avenue for ROP that could potentially be used in combination with anti-VEGF treatments. Since TLR4 mediates inflammation and neurodegeneration through pathways that may be independent of or upstream to VEGF—such as the aforementioned MAPK-mediated signaling—a dual approach could potentially enhance therapeutic efficacy and neuroprotection while reducing the required dosage and associated side effects of anti-VEGF agents.

In alignment with previous research examining TLR4's role in neuroinflammation across various disease models [[21], [22], [23], [24]], our study contributes to understanding TLR4's specific impact on retinal pathology in OIR. He et al. found that TLR2/4 double deficiency prevented hyperoxia-induced retinal vessel regression [25]. They also identified IL-17A as a key mediator of TLR2/4 signaling that promotes inflammation and inhibits revascularization. These findings support the concept that TLR4, potentially in concert with TLR2, can influence both the degenerative and regenerative phases of OIR. Notably, investigations into other retinal neurodegeneration models, including glaucoma and optic nerve injuries, have demonstrated that TLR4 knockdown offers neuroprotective effects, suggesting a potential therapeutic target for preserving retinal integrity in at-risk populations, particularly in premature infants susceptible to ROP [26,27].

Furthermore, our study uniquely demonstrates that TLR4 knockout not only inhibits pathological neovascularization but also significantly suppresses neuronal cell death in the OIR model. This neuroprotective effect represents a previously unrecognized dimension of TLR4's involvement in ROP pathogenesis. This finding is particularly important given the long-term visual consequences of neuronal loss in ROP.

I hypothesize that the neuroprotective effect observed in TLR4-knockout mice is mediated, at least in part, by the suppression of microglial activation and the subsequent reduction in the phagocytosis of retinal ganglion cells (RGCs). It is well established that activated microglia phagocytose stressed or dying neurons [28], and TLR4 activation serves as a primary driver of this microglial response [29]. By demonstrating that TLR4 deficiency mitigates both pathological angiogenesis and neuronal death, our study suggests that TLR4 acts as a pivotal regulator of both vascular and neurodegenerative processes in OIR. This modulation likely occurs through its influence on microglial activity and the consequent impact on RGC survival, providing a novel insight into the complex mechanisms underlying ROP pathogenesis.

Based on our findings and existing evidence, TLR4 signaling appears to exert multi-cellular effects within the retina, involving microglia, endothelial cells, and potentially the RGCs themselves. Consequently, TLR4-dependent inflammation may play a multifaceted role in orchestrating vascular remodeling and neurodegeneration. In particular, TLR4 activation is classically associated with the M1 polarization of macrophages and microglia, which promotes pro-inflammatory responses [30]. Therefore, the reduction in neovascularization and neuronal loss observed in TLR4-deficient mice may indicate a shift toward M2 polarization—a state generally linked to tissue repair and the resolution of aberrant angiogenesis [31]. Future investigations utilizing flow cytometry to quantify M1 and M2 populations in TLR4-deficient OIR retinas would be valuable for identifying cell-specific therapeutic targets.

Furthermore, it is crucial to clarify whether the loss of TLR4 impacts physiological angiogenesis. In our normoxic control group, the retinal vasculature of TLR4−/− mice appeared qualitatively normal at P19, exhibiting complete peripheral vascularization without detectable developmental defects. This observation aligns with previous reports indicating that TLR4 deficiency does not alter baseline retinal vascular patterns, vessel density, or developmental kinetics in neonatal mice [32]. The fact that TLR4 expression was nearly undetectable under normoxic conditions but markedly upregulated in microglia under OIR stress suggests that TLR4 primarily modulates pathological inflammatory responses rather than driving physiological developmental growth. Nevertheless, although global knockout mice provide a strong foundation for understanding the role of TLR4, they do not allow for the dissection of cell-specific contributions. Future research employing conditional knockout models (e.g., Cre-loxP systems) to target specific populations such as microglia, astrocytes, or endothelial cells will be essential to pinpoint the primary drivers of TLR4-mediated pathology. Additionally, a detailed time-course analysis of early developmental stages (e.g., P6–P12) and the kinetics of physiological revascularization would provide further clarity on the therapeutic window and safety profile of TLR4 modulation.

It's worth noting that in this study, I focused on vessel filling with FITC-dextran as a marker of retinal vascular angiogenesis. Comparing the intensity of blood vessels may not provide an accurate comparison, as it includes both normal and abnormal vessels. However, in this study, I utilized ImageJ to quantify neovascular tufts, allowing us to focus on a comparative analysis of abnormal vessels. In the future, direct quantification of vaso-obliteration and leakage will be essential for a more detailed understanding of retinal pathology, and the development of analytical software will be desirable. I plan to investigate these further in future studies.

Despite the significant findings, our study has several limitations. The OIR model, while widely used, does not fully recapitulate the complex multifactorial nature of human ROP, which involves additional factors such as prematurity, genetics, and systemic complications. Furthermore, while I demonstrated a strong association between TLR4 and OIR pathology, the precise molecular mechanisms downstream of TLR4 activation responsible for the observed angiogenic and neuroprotective effects require further elucidation. Our current investigation focused on gene expression at P19 and neuronal survival at P47; however, a more detailed time-course analysis of protein expression and cell-specific TLR4 signaling pathways would provide a deeper understanding. Lastly, the use of global TLR4-knockout mice prevents us from dissecting the cell-specific contributions of TLR4 to OIR pathogenesis, which could be addressed in future studies using conditional knockout models.

## 5 Conclusion

In conclusion, our study provides valuable insights into the intricate interplay among TLR4 signaling, angiogenesis, and neuronal cell survival within the context of Retinopathy of Prematurity (ROP). While these findings lay a crucial foundation, I acknowledge the need for further mechanistic studies to fully substantiate our conclusions and precisely delineate the molecular pathways modulated by TLR4. Future research will therefore focus on unraveling these detailed mechanisms and exploring their clinical implications for retinal diseases.

Critically, current ROP treatment predominantly relies on laser photocoagulation. This procedure, performed with an eyelid speculum on often intubated premature infants, is inherently highly invasive and profoundly stressful for these vulnerable patients. Consequently, the potential to achieve normal retinal vascular development prenatally by knocking down (KD) TLR4 represents a truly remarkable therapeutic advancement. Such a breakthrough would not only liberate premature infants from painful and stressful invasive treatments but also significantly alleviate the immense burden and emotional stress experienced by both the infants and their parents. Ultimately, this approach promises to enhance vision outcomes and dramatically improve the quality of life for affected individuals within this highly vulnerable population, marking it as an excellent and highly desirable therapeutic strategy.

## Formatting of funding sources

This research did not receive any specific grant from funding agencies in the public, commercial, or not-for-profit sectors.

## Declaration of competing interest

None. The authors have no conflicts of interest to declare.

## Data Availability

Data will be made available on request.
